# 3-Bromo-*N*-(3,5-di-*tert*-butyl­phen­yl)propanamide

**DOI:** 10.1107/S1600536814012094

**Published:** 2014-06-14

**Authors:** Anwar Abo-Amer, Mahmoud Al-Refai, Richard J. Puddephatt, Basem F. Ali

**Affiliations:** a Department of Chemistry, Al al-Bayt University, Mafraq 25113, Jordan; b Department of Chemistry, University of Western Ontario, London, N6A 5B7, Canada

## Abstract

The title compound, C_17_H_26_BrNO, exhibits a small twist between the amide residue and the benzene ring [C—N—C—C torsion angle = 29.4 (5)°]. In the crystal, the amido NH group is involved in N—H⋯O hydrogen bonding, which connects mol­ecules into chains parallel to the *c* axis.

## Related literature   

For the related structure of a derivative with an alkyl-*N*-aryl substituent, see: Palakshamurthy *et al.* (2014 ▶), with an alkyl-*N*-phenyl­sulfonyl substituent, see: Shakuntala *et al.* (2011 ▶) and with a chloro-*N*-phenyl substituent, see: Betz *et al.* (2011 ▶). For details of the synthesis, see: Bentiss & Lagrenée (1999 ▶); Hill *et al.* (2007 ▶).
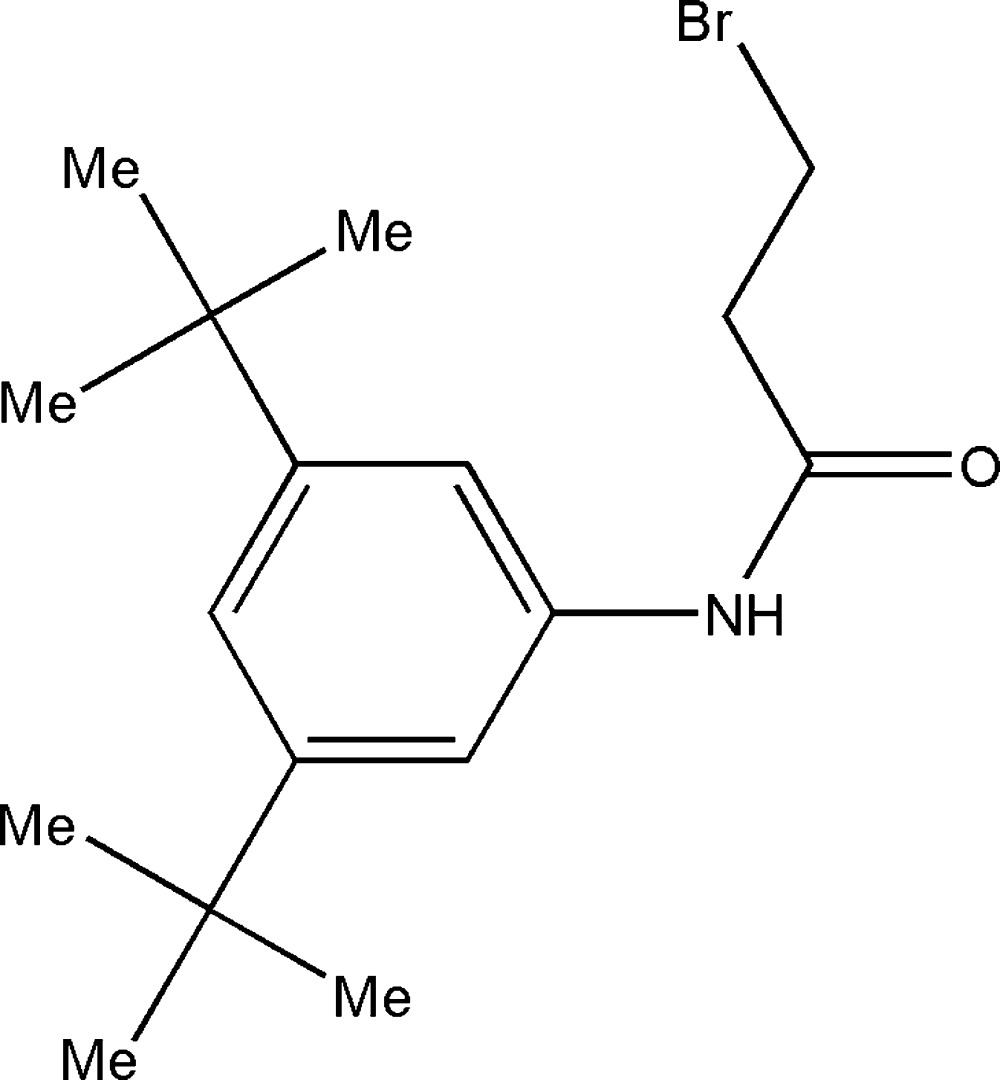


## Experimental   

### 

#### Crystal data  

C_17_H_26_BrNO
*M* *_r_* = 340.30
Monoclinic, 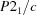

*a* = 15.666 (2) Å
*b* = 11.4885 (16) Å
*c* = 9.7829 (14) Å
β = 97.436 (4)°
*V* = 1745.9 (4) Å^3^
*Z* = 4
Mo *K*α radiation
μ = 2.35 mm^−1^
*T* = 150 K
0.40 × 0.20 × 0.11 mm

#### Data collection  

Bruker APEXII CCD diffractometer
Absorption correction: multi-scan (*SADABS*; Bruker, 2013 ▶) *T*_min_ = 0.455, *T*_max_ = 0.789
21557 measured reflections
4005 independent reflections
2738 reflections with *I* > 2σ(*I*)
*R*_int_ = 0.055

#### Refinement  

*R*[*F* ^2^ > 2σ(*F* ^2^)] = 0.050
*wR*(*F* ^2^) = 0.145
*S* = 1.04
4005 reflections
187 parameters
H-atom parameters constrained
Δρ_max_ = 1.08 e Å^−3^
Δρ_min_ = −0.65 e Å^−3^

### 

Data collection: *APEX2* (Bruker, 2013 ▶); cell refinement: *SAINT* (Bruker, 2013 ▶); data reduction: *SAINT*; program(s) used to solve structure: *SIR92* (Altomare *et al.*, 1994 ▶); program(s) used to refine structure: *SHELXL2013* (Sheldrick, 2008 ▶); molecular graphics: *CrystalMaker* (CrystalMaker, 2014 ▶); software used to prepare material for publication: local programs.

## Supplementary Material

Crystal structure: contains datablock(s) I, New_Global_Publ_Block. DOI: 10.1107/S1600536814012094/nk2223sup1.cif

Structure factors: contains datablock(s) I. DOI: 10.1107/S1600536814012094/nk2223Isup2.hkl

Supporting information file. DOI: 10.1107/S1600536814012094/nk2223Isup3.cml

CCDC reference: 1005148

Additional supporting information:  crystallographic information; 3D view; checkCIF report

## Figures and Tables

**Table 1:** Hydrogen-bond geometry (Å, °)

*D*—H⋯*A*	*D*—H	H⋯*A*	*D*⋯*A*	*D*—H⋯*A*
N1—H1*C*⋯O1^i^	0.88	2.01	2.889 (3)	174

Symmetry code: (i) 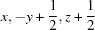.

**Fig. 1.**
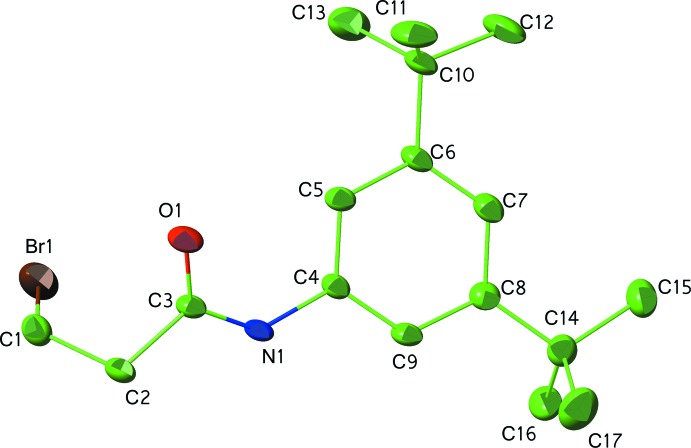
The molecular structure of the title compound, with atom labels and anisotropic displacement ellipsoids (drawn at 50% probability level).

**Fig. 2.**
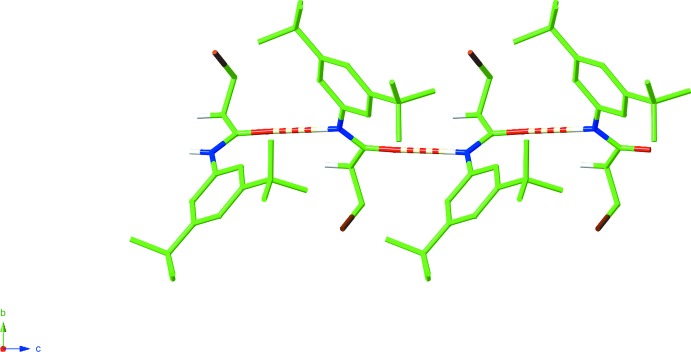
Partial molecular packing, showing the chains, parallel to c axis, formed via N—H···O hydrogen bonding (multi-rendered cylinders).

## supplementary crystallographic information

### S1. Comment

The title compound C_17_H_25_BrNO, is a brominated derivative of a secondary amide bearing a di-*tert*-butylbenzene ring. It exhibits a small twist between the amide residue and benzene ring [the C3—N1—C4—C5 torsion angle = 29.5 (4)°]. The N—H and C=O bonds are *anti* to each other (Fig. 1), as observed in many other derivatives (Shakuntala *et al.*, 2011). In the structure, bond distances and angles are within normal range (Table 1) and comparable to reported values in amide derivatives (Palakshamurthy *et al.*, 2014, Betz *et al.*, 2011). The torsion angle of C3—N1—C4—C9 and C3—N1—C4—C5 are -153.2 (3)° and 29.5 (4)°, respectively. The amido NH group is involved in N—H···O [2.01 Å] hydrogen bonding, which connects molecules into chains parallel to *c* axis (Fig. 2).

### S2. Experimental

Synthesis of (2,5-Bis(2-pyridyl)-1,3,4-oxadiazole)dimethylplatinum(II), [PtMe_2_(ox)]

A mixture of [Pt_2_Me_4_(µ-SMe_2_)_2_] (50 mg, 0.087 mmol)) (Hill *et al.*, 2007) and ox (ox = 2,5-bis(2-pyridyl)-1,3,4-oxadiazole) (38 mg, 0.170Dr mmol) (Bentiss and Lagrenée, 1999) in dry ether (10 ml) was stirred for 1 h. A red precipitate resulted. The precipitate was isolated and washed with acetone (3 × 5 ml). The product was recrystallized from CH_2_Cl_2_. A yellow solid was produced and dried *in vacuo*.

The title compound was crystallized unintentionally from the reaction mixture of the complex (2,5-Bis(2-pyridyl)-1,3,4-oxadiazole)dimethylplatinum(II), [PtMe_2_(ox)] (0.05 g, 0.112 mmol) and, the commercially available *N*-(3,5-di-*tert*-butylphenyl)-3-bromopropanamide (0.052 g, 0.129 mmol) in acetone (15 ml) was stirred for 5 h at room temperature. The reaction color changed to yellow suspension. The solvent was evaporated under vacuum and the resulting solid was washed with water (2 × 10 ml) and pentane (3 × 10 ml). The isolated yellow solid is highly soluble in CH_2_Cl_2_ solvent, which was dried under high vacuum. Yield 87%. A suitable crystal for X-ray diffraction analysis was selected for data collection.

### S3. Refinement

The hydrogen atoms were introduced at idealized positions and were allowed to ride on the parent atom, with C—H = 0.95–0.99 Å and N—H = 0.88 Å and *U*iso(H) = 1.2–1.5*U*eq(C,*N*).

### Crystal data

**Table:**

C_17_H_26_BrNO	*F*(000) = 712
*M**_r_* = 340.30	*D*_x_ = 1.295 Mg m^−^^3^
Monoclinic, *P*2_1_/*c*	Mo *K*α radiation, λ = 0.71073 Å
*a* = 15.666 (2) Å	Cell parameters from 5619 reflections
*b* = 11.4885 (16) Å	θ = 2.2–26.0°
*c* = 9.7829 (14) Å	µ = 2.35 mm^−^^1^
β = 97.436 (4)°	*T* = 150 K
*V* = 1745.9 (4) Å^3^	Needle, colourless
*Z* = 4	0.40 × 0.20 × 0.11 mm

### Data collection

**Table:**

Bruker APEXII CCD diffractometer	2738 reflections with *I* > 2σ(*I*)
φ and ω scans	*R*_int_ = 0.055
Absorption correction: multi-scan (*SADABS*; Bruker, 2013)	θ_max_ = 27.6°, θ_min_ = 2.2°
*T*_min_ = 0.455, *T*_max_ = 0.789	*h* = −20→19
21557 measured reflections	*k* = −14→14
4005 independent reflections	*l* = −8→12

### Refinement

**Table:**

Refinement on *F*^2^	0 restraints
Least-squares matrix: full	Hydrogen site location: inferred from neighbouring sites
*R*[*F*^2^ > 2σ(*F*^2^)] = 0.050	H-atom parameters constrained
*wR*(*F*^2^) = 0.145	*w* = 1/[σ^2^(*F*_o_^2^) + (0.0743*P*)^2^ + 1.5001*P*] where *P* = (*F*_o_^2^ + 2*F*_c_^2^)/3
*S* = 1.04	(Δ/σ)_max_ < 0.001
4005 reflections	Δρ_max_ = 1.08 e Å^−^^3^
187 parameters	Δρ_min_ = −0.65 e Å^−^^3^

### Special details

**Table:**

Geometry. All esds (except the esd in the dihedral angle between two l.s. planes) are estimated using the full covariance matrix. The cell esds are taken into account individually in the estimation of esds in distances, angles and torsion angles; correlations between esds in cell parameters are only used when they are defined by crystal symmetry. An approximate (isotropic) treatment of cell esds is used for estimating esds involving l.s. planes.

### Fractional atomic coordinates and isotropic or equivalent isotropic displacement parameters (Å^2^)

**Table:**

	*x*	*y*	*z*	*U*_iso_*/*U*_eq_	
Br1	0.58935 (3)	0.54272 (3)	0.85526 (5)	0.04516 (17)	
N1	0.61402 (17)	0.2102 (2)	0.8672 (2)	0.0205 (6)	
H1C	0.5990	0.2131	0.9508	0.025*	
O1	0.57853 (15)	0.2805 (2)	0.6495 (2)	0.0292 (6)	
C1	0.4854 (2)	0.4604 (3)	0.7774 (4)	0.0303 (8)	
H1A	0.4344	0.4974	0.8092	0.036*	
H1B	0.4790	0.4653	0.6756	0.036*	
C2	0.4901 (2)	0.3353 (3)	0.8208 (3)	0.0234 (7)	
H2A	0.4358	0.2957	0.7840	0.028*	
H2B	0.4961	0.3308	0.9227	0.028*	
C3	0.56518 (19)	0.2730 (3)	0.7699 (3)	0.0201 (6)	
C4	0.68642 (19)	0.1401 (3)	0.8510 (3)	0.0201 (6)	
C5	0.74057 (19)	0.1639 (3)	0.7525 (3)	0.0219 (7)	
H5	0.7282	0.2270	0.6904	0.026*	
C6	0.81286 (19)	0.0950 (3)	0.7452 (3)	0.0226 (7)	
C7	0.8718 (2)	0.1217 (3)	0.6355 (3)	0.0267 (7)	
C8	0.8922 (3)	0.2519 (4)	0.6334 (5)	0.0476 (11)	
H8A	0.9299	0.2674	0.5631	0.071*	
H8B	0.9211	0.2757	0.7240	0.071*	
H8C	0.8386	0.2959	0.6118	0.071*	
C9	0.8285 (2)	0.0018 (3)	0.8365 (3)	0.0240 (7)	
H9	0.8774	−0.0460	0.8308	0.029*	
C10	0.7749 (2)	−0.0236 (3)	0.9359 (3)	0.0222 (7)	
C11	0.7922 (2)	−0.1243 (3)	1.0380 (3)	0.0263 (7)	
C12	0.7155 (3)	−0.2089 (4)	1.0182 (5)	0.0467 (10)	
H12A	0.6632	−0.1682	1.0367	0.070*	
H12B	0.7267	−0.2745	1.0820	0.070*	
H12C	0.7077	−0.2378	0.9232	0.070*	
C13	0.8033 (3)	−0.0751 (3)	1.1850 (4)	0.0364 (9)	
H13A	0.8532	−0.0231	1.1975	0.055*	
H13B	0.8122	−0.1393	1.2514	0.055*	
H13C	0.7515	−0.0316	1.2001	0.055*	
C14	0.8740 (2)	−0.1920 (3)	1.0187 (4)	0.0355 (9)	
H14A	0.8691	−0.2226	0.9245	0.053*	
H14B	0.8813	−0.2567	1.0844	0.053*	
H14C	0.9238	−0.1400	1.0349	0.053*	
C15	0.7037 (2)	0.0485 (3)	0.9422 (3)	0.0218 (6)	
H15	0.6666	0.0344	1.0100	0.026*	
C16	0.8254 (2)	0.0852 (4)	0.4948 (4)	0.0385 (9)	
H16A	0.7720	0.1299	0.4744	0.058*	
H16B	0.8119	0.0020	0.4961	0.058*	
H16C	0.8626	0.1004	0.4236	0.058*	
C17	0.9571 (2)	0.0552 (4)	0.6613 (4)	0.0412 (10)	
H17A	0.9459	−0.0285	0.6509	0.062*	
H17B	0.9849	0.0712	0.7549	0.062*	
H17C	0.9949	0.0802	0.5945	0.062*	

### Atomic displacement parameters (Å^2^)

**Table:**

	*U*^11^	*U*^22^	*U*^33^	*U*^12^	*U*^13^	*U*^23^
Br1	0.0416 (3)	0.0352 (2)	0.0631 (3)	−0.00389 (17)	0.0234 (2)	−0.00846 (19)
N1	0.0228 (13)	0.0298 (15)	0.0107 (12)	0.0051 (11)	0.0087 (10)	−0.0011 (10)
O1	0.0302 (13)	0.0479 (15)	0.0109 (10)	0.0089 (11)	0.0083 (9)	0.0029 (10)
C1	0.0244 (17)	0.038 (2)	0.0295 (18)	0.0105 (15)	0.0071 (14)	0.0062 (15)
C2	0.0170 (15)	0.0324 (18)	0.0221 (16)	0.0020 (13)	0.0082 (12)	−0.0002 (13)
C3	0.0179 (15)	0.0277 (17)	0.0157 (15)	−0.0025 (12)	0.0057 (12)	−0.0035 (12)
C4	0.0169 (15)	0.0278 (17)	0.0164 (14)	−0.0003 (12)	0.0055 (12)	−0.0042 (13)
C5	0.0202 (16)	0.0322 (17)	0.0142 (14)	0.0000 (13)	0.0059 (12)	0.0012 (13)
C6	0.0182 (15)	0.0329 (18)	0.0175 (15)	−0.0009 (13)	0.0053 (12)	−0.0063 (13)
C7	0.0209 (16)	0.039 (2)	0.0216 (16)	0.0031 (14)	0.0092 (13)	−0.0050 (14)
C8	0.044 (3)	0.047 (3)	0.059 (3)	−0.0080 (19)	0.034 (2)	−0.004 (2)
C9	0.0204 (16)	0.0315 (16)	0.0205 (16)	0.0034 (13)	0.0040 (13)	−0.0070 (14)
C10	0.0228 (16)	0.0280 (18)	0.0158 (15)	−0.0019 (13)	0.0027 (12)	−0.0049 (13)
C11	0.0258 (17)	0.0294 (18)	0.0236 (16)	0.0040 (14)	0.0021 (13)	0.0014 (14)
C12	0.044 (2)	0.037 (2)	0.058 (3)	−0.0040 (18)	0.001 (2)	0.012 (2)
C13	0.045 (2)	0.044 (2)	0.0216 (17)	0.0137 (17)	0.0070 (16)	0.0071 (16)
C14	0.042 (2)	0.036 (2)	0.0290 (19)	0.0125 (17)	0.0032 (16)	0.0002 (16)
C15	0.0222 (16)	0.0289 (17)	0.0158 (14)	−0.0024 (13)	0.0084 (12)	−0.0026 (13)
C16	0.036 (2)	0.061 (3)	0.0204 (17)	−0.0049 (18)	0.0109 (15)	−0.0013 (17)
C17	0.028 (2)	0.064 (3)	0.035 (2)	0.0075 (18)	0.0159 (16)	−0.0010 (19)

### Geometric parameters (Å, º)

**Table:**

Br1—C1	1.951 (4)	C9—C10	1.396 (5)
N1—C3	1.350 (4)	C9—H9	0.9500
N1—C4	1.416 (4)	C10—C15	1.397 (4)
N1—H1C	0.8800	C10—C11	1.529 (5)
O1—C3	1.226 (4)	C11—C14	1.531 (5)
C1—C2	1.498 (5)	C11—C13	1.534 (5)
C1—H1A	0.9900	C11—C12	1.538 (5)
C1—H1B	0.9900	C12—H12A	0.9800
C2—C3	1.515 (4)	C12—H12B	0.9800
C2—H2A	0.9900	C12—H12C	0.9800
C2—H2B	0.9900	C13—H13A	0.9800
C4—C15	1.384 (4)	C13—H13B	0.9800
C4—C5	1.391 (4)	C13—H13C	0.9800
C5—C6	1.391 (4)	C14—H14A	0.9800
C5—H5	0.9500	C14—H14B	0.9800
C6—C9	1.396 (5)	C14—H14C	0.9800
C6—C7	1.534 (4)	C15—H15	0.9500
C7—C8	1.530 (6)	C16—H16A	0.9800
C7—C16	1.530 (5)	C16—H16B	0.9800
C7—C17	1.531 (5)	C16—H16C	0.9800
C8—H8A	0.9800	C17—H17A	0.9800
C8—H8B	0.9800	C17—H17B	0.9800
C8—H8C	0.9800	C17—H17C	0.9800
			
C3—N1—C4	127.9 (2)	C9—C10—C15	117.6 (3)
C3—N1—H1C	116.1	C9—C10—C11	122.7 (3)
C4—N1—H1C	116.1	C15—C10—C11	119.7 (3)
C2—C1—Br1	110.3 (2)	C10—C11—C14	112.6 (3)
C2—C1—H1A	109.6	C10—C11—C13	108.8 (3)
Br1—C1—H1A	109.6	C14—C11—C13	107.9 (3)
C2—C1—H1B	109.6	C10—C11—C12	109.0 (3)
Br1—C1—H1B	109.6	C14—C11—C12	108.4 (3)
H1A—C1—H1B	108.1	C13—C11—C12	110.0 (3)
C1—C2—C3	111.8 (3)	C11—C12—H12A	109.5
C1—C2—H2A	109.2	C11—C12—H12B	109.5
C3—C2—H2A	109.2	H12A—C12—H12B	109.5
C1—C2—H2B	109.2	C11—C12—H12C	109.5
C3—C2—H2B	109.2	H12A—C12—H12C	109.5
H2A—C2—H2B	107.9	H12B—C12—H12C	109.5
O1—C3—N1	124.3 (3)	C11—C13—H13A	109.5
O1—C3—C2	121.2 (3)	C11—C13—H13B	109.5
N1—C3—C2	114.5 (3)	H13A—C13—H13B	109.5
C15—C4—C5	120.7 (3)	C11—C13—H13C	109.5
C15—C4—N1	116.9 (3)	H13A—C13—H13C	109.5
C5—C4—N1	122.3 (3)	H13B—C13—H13C	109.5
C4—C5—C6	119.9 (3)	C11—C14—H14A	109.5
C4—C5—H5	120.1	C11—C14—H14B	109.5
C6—C5—H5	120.1	H14A—C14—H14B	109.5
C5—C6—C9	118.7 (3)	C11—C14—H14C	109.5
C5—C6—C7	119.4 (3)	H14A—C14—H14C	109.5
C9—C6—C7	121.9 (3)	H14B—C14—H14C	109.5
C8—C7—C16	109.3 (3)	C4—C15—C10	120.9 (3)
C8—C7—C17	108.2 (3)	C4—C15—H15	119.6
C16—C7—C17	108.3 (3)	C10—C15—H15	119.6
C8—C7—C6	110.5 (3)	C7—C16—H16A	109.5
C16—C7—C6	108.4 (3)	C7—C16—H16B	109.5
C17—C7—C6	112.1 (3)	H16A—C16—H16B	109.5
C7—C8—H8A	109.5	C7—C16—H16C	109.5
C7—C8—H8B	109.5	H16A—C16—H16C	109.5
H8A—C8—H8B	109.5	H16B—C16—H16C	109.5
C7—C8—H8C	109.5	C7—C17—H17A	109.5
H8A—C8—H8C	109.5	C7—C17—H17B	109.5
H8B—C8—H8C	109.5	H17A—C17—H17B	109.5
C6—C9—C10	122.3 (3)	C7—C17—H17C	109.5
C6—C9—H9	118.9	H17A—C17—H17C	109.5
C10—C9—H9	118.9	H17B—C17—H17C	109.5
			
Br1—C1—C2—C3	−61.5 (3)	C9—C6—C7—C17	−13.4 (4)
C4—N1—C3—O1	−1.8 (5)	C5—C6—C9—C10	−0.9 (5)
C4—N1—C3—C2	178.0 (3)	C7—C6—C9—C10	−179.6 (3)
C1—C2—C3—O1	−48.6 (4)	C6—C9—C10—C15	−0.2 (5)
C1—C2—C3—N1	131.6 (3)	C6—C9—C10—C11	−179.0 (3)
C3—N1—C4—C15	−153.2 (3)	C9—C10—C11—C14	0.3 (4)
C3—N1—C4—C5	29.4 (5)	C15—C10—C11—C14	−178.5 (3)
C15—C4—C5—C6	0.0 (5)	C9—C10—C11—C13	119.9 (3)
N1—C4—C5—C6	177.3 (3)	C15—C10—C11—C13	−58.9 (4)
C4—C5—C6—C9	1.0 (5)	C9—C10—C11—C12	−120.1 (4)
C4—C5—C6—C7	179.7 (3)	C15—C10—C11—C12	61.1 (4)
C5—C6—C7—C8	47.2 (4)	C5—C4—C15—C10	−1.2 (5)
C9—C6—C7—C8	−134.1 (3)	N1—C4—C15—C10	−178.6 (3)
C5—C6—C7—C16	−72.6 (4)	C9—C10—C15—C4	1.3 (5)
C9—C6—C7—C16	106.1 (4)	C11—C10—C15—C4	−179.9 (3)
C5—C6—C7—C17	167.9 (3)		

### Hydrogen-bond geometry (Å, º)

**Table:**

*D*—H···*A*	*D*—H	H···*A*	*D*···*A*	*D*—H···*A*
N1—H1*C*···O1^i^	0.88	2.01	2.889 (3)	174
C5—H5···O1	0.95	2.41	2.931 (4)	115

Symmetry code: (i) *x*, −*y*+1/2, *z*+1/2.
